# Righting Reflex Predicts Long-Term Histological and Behavioral Outcomes in a Closed Head Model of Traumatic Brain Injury

**DOI:** 10.1371/journal.pone.0161053

**Published:** 2016-09-22

**Authors:** Natalia M. Grin’kina, Yang Li, Margalit Haber, Michael Sangobowale, Elena Nikulina, Charm Le’Pre, Alexander M. El Sehamy, Rachelle Dugue, Johnson S. Ho, Peter J. Bergold

**Affiliations:** 1 Program in Neural and Behavioral Science, SUNY-Downstate Medical Center, Brooklyn, NY, United States of America; 2 Robert F. Furchgott Center for Neural Science, SUNY-Downstate Medical Center, Brooklyn, NY, United States of America; 3 Department of Physiology and Pharmacology SUNY-Downstate Medical Center, 450 Clarkson Avenue, Brooklyn, NY, 11203, United States of America; University of Florida, UNITED STATES

## Abstract

Blunt impact produces a heterogeneous brain injury in people and in animal models of traumatic brain injury. We report that a single closed head impact to adult C57/BL6 mice produced two injury syndromes (CHI-1 and CHI-2). CHI-1 mice spontaneously reinitiated breathing after injury while CHI-2 mice had prolonged apnea and regained breathing only after cardiopulmonary resuscitation and supplementation of 100% O_2_. The CHI-1 group significantly regained righting reflex more rapidly than the CHI-2 group. At 7 days post-injury, CHI-1, but not CHI-2 mice, acquired but had no long-term retention of an active place avoidance task. The behavioral deficits of CHI-1 and CHI-2 mice were retained one-month after the injury. CHI-1 mice had loss of hippocampal neurons and localized white matter injury at one month after injury. CHI-2 had a larger loss of hippocampal neurons and more widespread loss of myelin and axons. High-speed videos made during the injury were followed by assessment of breathing and righting reflex. These videos show that CHI-2 mice experienced a larger vertical g-force than CHI-1 mice. Time to regain righting reflex in CHI-2 mice significantly correlated with vertical g-force. Thus, physiological responses occurring immediately after injury can be valuable surrogate markers of subsequent behavioral and histological deficits.

## Introduction

Blunt impact to the head is the most common cause of traumatic brain injury[1]. Rodent models of blunt impact TBI include closed and open head models. In closed head models, the head can move while the skull remains intact. In open head models, the head is immobilized and the brain is directly injured through a craniotomy[2, 3]. The craniotomy, however, damages the skull and underlying tissues and produces inflammation[4]. Even thinning of the skull without exposing the meninges produces vascular changes, inflammation and cell death[5]. Open head models have the advantage of producing a relatively uniform injury by directly deforming the brain[2, 4, 6]. Closed head models require greater force to injure the brain since the skull remains intact. In mice, the force needed to reliably produce behavioral deficits in surviving mice frequently produced skull fractures [6]. Some investigators needed to hit the skull of the mouse multiple times to produce behavioral deficits on purely spatial tasks such as the Morris water maze or Barnes maze [7,8]. Behavioral deficits, however, did arise using an active place avoidance task after a single closed head impact. The deficits on the active place avoidance task that arose after a single impact likely result from a need for both hippocampi and their commissural connections to acquire the task [9, 10]. TBI models frequently damage the hippocampi and their commissural connections. In contrast, the Morris water maze or Barnes maze can be acquired with only one functional hippocampus [10–12].

Rotational acceleration/deceleration is a major cause of brain damage in clinical TBI [2]. Few rodent models reproduce this rotational acceleration/ deceleration because the head is either fixed or the impact is directed at the midline of the skull[2, 13]. We examined the effects of a lateral strike of the scalp of mice with an electromagnetically controlled piston. We found that mice could be divided into CHI-1 and CHI-2 groups according to the time needed to restore the righting reflex. Head movements of mice were then analyzed during and after the impact. We also found that the CHI-1 and CHI-2 groups had long-lasting differences in behavior and histology.

## Material and Methods

### Closed head injury

Closed head injury was done on a modified Kopf stereotaxic apparatus by placing a single 0.5- inch sheet of polyurethane foam on the bed of the adaptor and two 0.5-inch sheets of polyurethane foam wrapped around the ear bar holders. All experiments were done on male C57/BL6 mice (15 to 17 weeks old, 26-28gr, Jackson Laboratories, Bar Harbor, Maine). Mice were randomly assigned to a sham-CHI or CHI group. A baseline weight was obtained prior to sham-CHI or CHI. The top of the head of the mouse was shaved. Deep anesthesia was induced for 2 minutes with isoflurane (3.5% in oxygen (1.0 L/min)) and maintained (3% in oxygen (1.0 L/min)) until immediately after the impact. The head was placed between the two padded ear bar holders that allowed the nose of the mouse to be inserted into an anesthetic gas nose cone (#39462950, Leica Microsystems, Buffalo Grove, IL). Rectal temperature was maintained using a circulating warm water heating pad whose temperature was controlled at 36.5–37.5°C (Kent Scientific, Torrington, CT). Closed head injury was produced using a 5.0 mm diameter impactor tip controlled by an electromagnetic impactor (Leica Microsystems, Buffalo Grove, IL). The impactor tip was placed 5mm lateral from the midline and 2mm caudal from the eyes, at a 10° angle and produced a single 6.3 m/s impact on the scalp to a depth of 3 mm with a 1-s dwell time. These coordinates produced an impact above the parietal lobe. Sham-injured mice had an identical treatment without the impact. The entire procedure was completed in less than 3 minutes. The breathing status of the mouse was assessed after injury. If a mouse did not spontaneously breath within 30 seconds, cardiopulmonary resuscitation was initiated with chest compressions at a rate of 150–160 per minute, while the mouse breathed 100% O_2_. The length of apnea in the sham-CHI and CHI-1 groups was not recorded. Recovery of righting reflex was assessed by placing the animal on its back and the time to right themselves was measured. Mice were then returned to their home cages.

This study was carried out in strict accordance with the recommendations in the Guide for the Care and Use of Laboratory Animals of the National Institutes of Health. The protocol was approved by the Institutional Animal Care and Use Committee of the State University of New York—Downstate Medical Center (Permit Number: 12–10351). All surgeries were performed under isoflurane anesthesia, and all efforts were made to minimize suffering.

### Biomechanical Measurements

To assess head motion during the impact, one midline marker was placed 2-mm caudal to the eyes, and two markers were placed 4-mm ipsilateral and contralateral to the midline marker. Movement of these markers was traced using a high-resolution digital imaging system (Epic Mysterium-X; Red Digital Cinema Professional, USA) at 2.80 msec/frame. Head displacement was measured from video frames using ImageJ and Manual Tracking plug-in (http://rsb.info.nih.gov/ij/plugins/track/track.html). Vertical, lateral, and angular (ipsilateral relative to midline) displacements were converted to vertical, lateral, and angular accelerations from the second derivative of the head displacement and the time elapsed per video frame. Division by the gravity constant (9.8) converted these accelerations into units of g-force (newtons/kg). Analysis of head motion began 4.8 msec before the impact and continued for 52.8 msec after impact.

### Motor assessment

A rotarod (Harvard Apparatus, Holliston, MA) assessed motor ability 1, 3, and 7 days after sham-CHI or CHI. Training consisted of a 5-minute training/habituation trial with the rod rotating at 4rpm followed by four 5-minute trials with the speed of the rotarod beginning at 4rpm and accelerating to 7rpm. Total time that the mice stayed on the rod over 4 trials analyzed motor ability. Between trials, mice were returned to their home cages for 5 minutes.

### Active place avoidance

Behavioral assessment protocol was done with modifications from Burghardt, et al. [14]. Behavioral assessments were done in a rectangular room (4m x 3m) containing a behavioral apparatus that consisted of a 40-cm diameter circular arena that rotated at 1 rpm. The room had prominent visual landmarks on the walls. A computer tracked the position of the mouse using a computer controlled infrared Firewire camera located 1.2 m above the arena. The signal from the camera was analyzed using a spot-tracker (BioSignal Group, Brooklyn, NY). The movement of the animal relative to the arena was calculated every 33 msec from the positions of the mouse and a light emitting diode that marked the outer part of the arena. During active place avoidance, the computer defined a 60° segment of the arena as a shock zone. Entry into the shock zone for 500msec triggered a shock of constant current (500 msec, 60 Hz, 0.2 mA) delivered through the grid floor. Additional shocks were administered every 1.5 s until the mouse left the shock zone. Track analysis software (Bio-Signal Group Corp., Brooklyn, NY) analyzed the movement of the mouse and the number of entries into the shock zone.

On day 7 after CHI or sham-CHI, mice were habituated to handling and the training environment. Mice first received a 10-minute habituation/open field session with the shock zone turned off. Total distance traveled was assessed. The mice then had 4 x 10-minute sessions of active place avoidance with an active shock zone. Total distance traveled, speed, linearity, shocks/entrance, number of shock zone entrances and time to first entrance were assessed. The mice had a 50-minute interval in their home cages between trials. On the following day, if the mice reduced the number of entrances on active place avoidance, they received an additional 4 x 10-minute sessions of conflict active place avoidance with a 50-minute intertrial interval. In conflict active place avoidance, the shock zone was rotated 180° from its original location on the previous day. Total distance traveled, speed, linearity, shocks/entrance, number of shock zone entrances and time to first entrance were assessed. Mice were returned to their home cages after testing.

### Histological analysis

One month after sham-CHI or CHI, mice were deeply anesthetized using isoflurane (3–5%) in oxygen (0.8 L/min) and fixed transcardially with paraformaldehyde (4%, w/v). The brains were imbedded in paraffin and parasagittal sections were prepared (HistoWiz, Inc., Brooklyn, NY). The sections were stained with SMI-312 that recognized all three neurofilament subunits (1:1000, Abcam), anti-NeuN (1:500, MAB377, Chemicon) or anti-MAP2 (1:1000, SMI-52, Abcam). Immunocomplexes were visualized with the appropriate fluorescent secondary antibody. The amount of immunofluorescence minus background was assessed in specific regions of interest using ImageJ v.1.48 software (rsbweb.nih.gov/ij/download.html). NeuN immunopositive cells (NeuN^+^) were counted in 500 μm of the pyramidal cell layer in the CA3 and CA1 regions or in 500 μm of the granule cell layer in the suprapyramidal blade of the dentate gyrus. NeuN^+^ cells in the hilus were counted between the suprapyramidal and infrapyramidal blades of the dentate gyrus.

Sections were also stained with kits for Luxol Fast Blue (LFB) or Bielschowsky's modified silver stain according to the manufacturer’s instructions (American Mastertech, Lodi, CA). The amount of LFB or silver staining was assayed in specific regions of interest using ImageJ software. To measure the LFB staining corresponding to myelin, LFB dye intensity in heavily myelinated regions of interest were subtracted from the unmyelinated stratum radiatum of the hippocampus [15].

### Statistical Analysis

Two-way mixed ANOVA compared the CHI-1, CHI-2, and sham-CHI groups (between-subjects factor) across trials or days (within-subjects factor) on active place avoidance, conflict active place avoidance, and rotarod. The distribution of entrances on the conflict active place avoidance dataset violated sphericity and was therefore subjected to the Greenhouse-Geisser correction. Time to 1st entrance was analyzed by one-way ANOVA for active place avoidance and by student’s t-test for conflict active place avoidance. One-way ANOVA was used to analyze group differences in histology. After using one-way or two-way ANOVA, pairwise comparisons were analyzed using the Tukey HSD post hoc test. Head motion was analyzed using two-way repeated measures ANOVA. Vertical g-force and time to regain righting reflex was compared using Spearman's rank correlation coefficient. All values are given as the mean ± the standard error of the mean. In all tests, statistical significance was set at 0.05.

## Results

### Mice can be stratified by righting reflex following closed head injury

The breathing status of mice was monitored immediately after sham-CHI (n = 9) or CHI (n = 22) until spontaneous breathing resumed. Sham-CHI mice began breathing within seconds after the end of anesthesia. One group of CHI mice, termed CHI-1, transiently stopped breathing but recovered spontaneous breathing within 30 seconds after the cessation of anesthesia (n = 9). In contrast, a second group termed CHI-2 (n = 13) did not begin breathing within 30s and required cardiopulmonary resuscitation and ventilation with 100% O_2_ until unassisted breathing resumed. The 30-second cut-off to administer cardiopulmonary resuscitation was chosen since 100% of mice died if resuscitation with 100% O_2_ did not begin within this time period (data not shown). CHI-2 with prolonged apnea did not survive if they were resuscitated without supplementation of 100% O_2_, suggesting that it was needed for their survival (data not shown). In this study, 55.1% of the injured mice were placed into the CHI-2 group and 44.9% were placed into the CHI-1 group.

**Fig 1:**
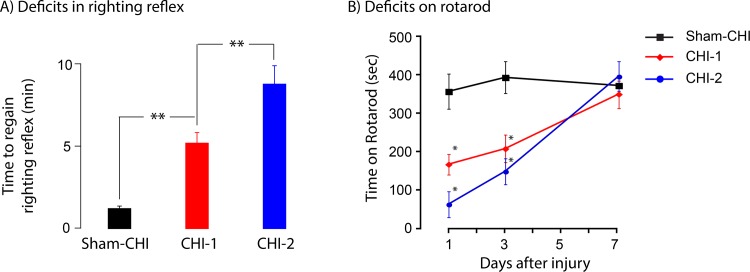
CHI-1 and CHI-2 mice have differing deficits in righting reflex and rotarod after closed head injury. Panel A, Assessment of righting reflex. Recovery of righting reflex differed among sham-CHI, CHI-1 and CHI-2 mice (ANOVA, F_2,30_ = 18.4, p < 0.001). Sham-CHI mice recovered righting reflex most rapidly followed by CHI-1 and then by CHI-2 (post hoc p<0.01). Panel B, One, three and seven days after either sham-CHI or CHI, mice received four 5-minute trials on the rotarod. Performance on the rotarod had a significant interaction between group and time (F_4,14_ = 5.03, p = 0.01). Rotarod performance also had a significant effect between-subjects across days F_2,7_ = 5.45, p = 0.04). The three groups significantly differed on the time on the rotarod on Days 1 (F_2,17_ = 10.25, p = 0.001), and 3 (F_2,17_ = 10.63, p = 0.001), but not on Day 7 (F_2,10_ = 0.42, p = 0.68). The performance of the sham-CHI group on Day 1 and 3 was significantly better than the CHI-1 and CHI-2 groups (post hocs, p = 0.001). There were no statistically significant differences in rotarod performance between conditions on Day 7 suggesting that the motor deficits at 1 and 3 days were transient.

Restoration of righting reflex was also assessed after injury (Fig 1A). The sham-CHI, CHI-1 and CHI-2 groups significantly differed in time elapsed before recovery of righting reflex. The sham-CHI group recovered most rapidly followed by the CHI-1 group and then the CHI-2 group (post hoc, p<0.01). Within one day after the injury, 9.1% of the CHI-2 mice died. Post-mortem examination of these mice showed the presumptive cause of death was cerebral hemorrhages underlying the impact site (data not shown). No fatalities occurred in the sham-CHI or CHI-1 groups.

### CHI-1 and CHI-2 mice have transient motor deficits

CHI-injured mice were divided into CHI-1 or CHI-2 groups based upon time to regain righting reflex. The motor ability of the sham-CHI (n = 4), CHI-1 (n = 4) and CHI-2 (n = 4) group were tested using a rotarod 1, 3 and 7 days after sham-CHI or CHI (Fig 1B). On days 1 and 3, the three groups differed significantly on the total time on the rotating rod. On day 7, all groups spent equal time on the rod suggest that CHI-1 and CHI-2 mice had motor deficits on days 1 and 3 that were absent on day 7. Transient motor deficits are common in animal models of TBI [2].

### CHI-1 and CHI-2 mice differ in behavioral deficits one week after injury

**Fig 2:**
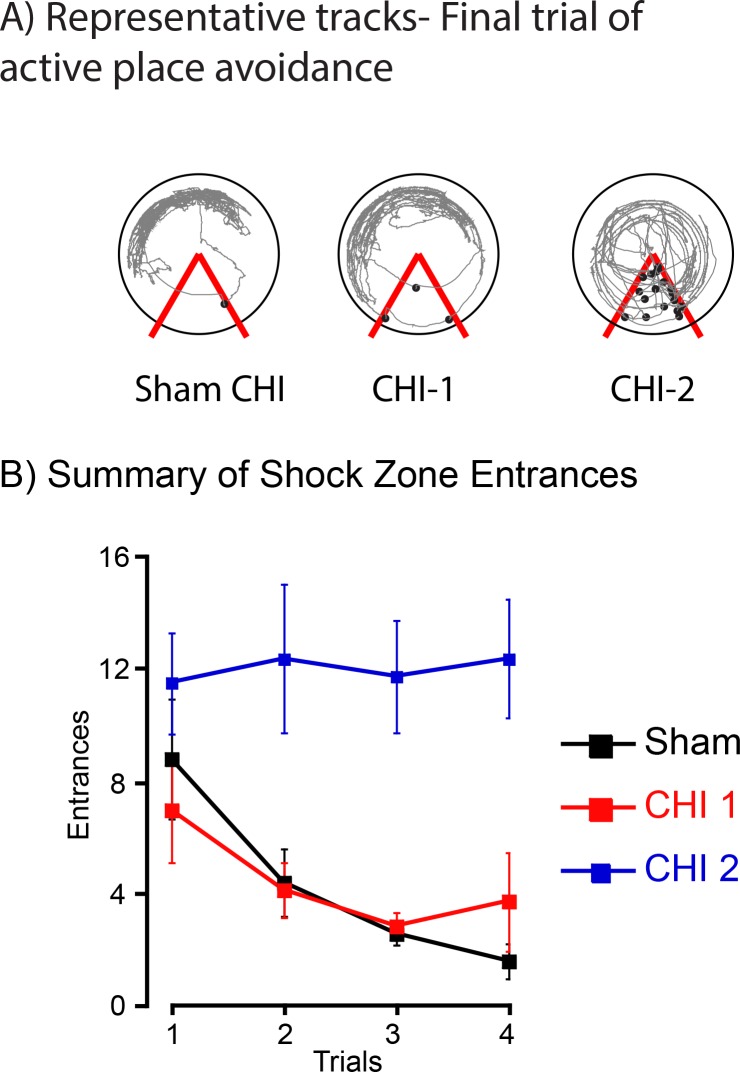
CHI-1, but not CHI-2 mice acquired an active place avoidance task. The sham-CHI, CHI- 1 or CHI-2 groups received four 10-minute trials with a 50-minute intertrial interval. Panel A, Representative tracks of the final trial of sham-CHI, CHI-1 and CHI-2 mice. The 60° stationary shock zone is shown in red. Black circles show the location of the mouse when being shocked. Panel B, Summary of total shock zone entrances during the 4 trials of active place avoidance. The number of shock zone entrances significantly differed on the basis of trial number for the Sham-CHI (F_3,12_ = 12.88, p < 0.0005) and the CHI-1 groups (F_3,18_ = 8.07, p = 0.001), but not for the CHI 2 group (F_3,21_ = 0.32, p = 0.81). Furthermore, there was a statistically significant interaction between group and trial number (F_6,51_ = 3.81, p < 0.003). The CHI-2 group had significantly more shock zone entrances than the sham-CHI or CHI-1 groups. In contrast, the sham-CHI and CHI-1 groups had a similar number of shock zone entrances (post hoc, p = 0.05) suggesting that the sham-CHI and CHI-1 groups, but not the CHI-2 group acquired the shock zone location.

At seven days after injury, the sham-CHI (n = 5), CHI-1 (n = 6) and CHI-2 (n = 5) groups were trained on an active place avoidance task. All three groups traveled a similar distance in the habituation/open field trial (Table 1). The mice were then tested with the shock zone activated. Representative tracks of the 4^th^ and final session show that sham-CHI and CHI-1 mice avoided the shock zone while CHI-2 mice did not (Fig 2). Analysis of the number of shock zone entrances showed a significant group effect with CHI-2 mice having more entrances than sham-CHI and CHI-1 mice. During active place avoidance, the sham-CHI and CHI-1 groups could lower the number of entrances while the CHI-2 group could not (Fig 2). These data strongly suggest that only the sham-CHI and CHI-1 groups acquired the shock zone location.

**Table 1:** Measurements of mouse behavior during behavioral testing.

Task	Parameter	Sham-CHI	CHI-1	CHI-2
Habituation	Total Distance	49.8 ± 2.0	46.4 ± 2.7	41.9 ± 3.0
Active Place Avoidance	Total Distance	21.9 ± 0.71	23.7 ± 1.3	26.1 ± 3.0
	Speed	3.65 ± 0.12	3.9 ± 0.17	4.35 ± 0.6
	Linearity	0.52 ± 0.02	0.51 ± 0.02	0.55 ± 0.03
	Shocks per Entrance	1.1 ± 0.05	1.1 ± 0.03	1.2 ± 0.06

During habituation, all three groups traveled a similar distance (F_2,15_ = 1.96, p > 0.1). During active place avoidance, there was no significant group differences on total distance traveled, (F_2,15_ = 1.78, p > 0.2), speed (F_2,15_ = 1.79, p > 0.2), linearity (F_2,15_ = 0.98, p > 0.4) or shocks per entrance (F_2,15_ = 1.57, p > 0.2).

Time to 1st entrance measures whether a mouse recalls a shock zone location from a previous trial[16]. Increasing time to 1st entrance requires avoiding the shock zone without the sensory cue of a foot shock. Sham-CHI mice had a significantly longer time to 1st entrance than CHI-1 or CHI-2 mice (sham-CHI, 500.6 ± 75.9 sec; CHI-1, 97.5 ± 38.0 sec; CHI-2, 32.0 ± 2.2 sec; ANOVA, F_2,15_ = 21.20, p > 0.001; post hoc, p < 0.0005). These data suggest that the three groups differed on active place avoidance since the sham-CHI group both acquired and retained the shock zone location, the CHI-1 group acquired but did not retain the shock zone location and the CHI-2 mice neither acquired nor retained the shock zone location.

The sham-CHI, CHI-1 and CHI-2 groups had similar motor ability 7 days after sham-CHI or CHI since there were no differences on the rotarod or in distance traveled, linearity or speed on active place avoidance (Fig 1B, Table 1). The three groups also had similar abilities to sense shock and exit the shock zone after being shocked since they did not differ in the numbers of shocks received for each shock zone entrance (Table 1). These data suggest that cognition and memory deficits underlie the behavioral differences among sham-CHI, CHI-1 and CHI-2 mice since the three groups retain similar sensory and motor abilities.

**Fig 3:**
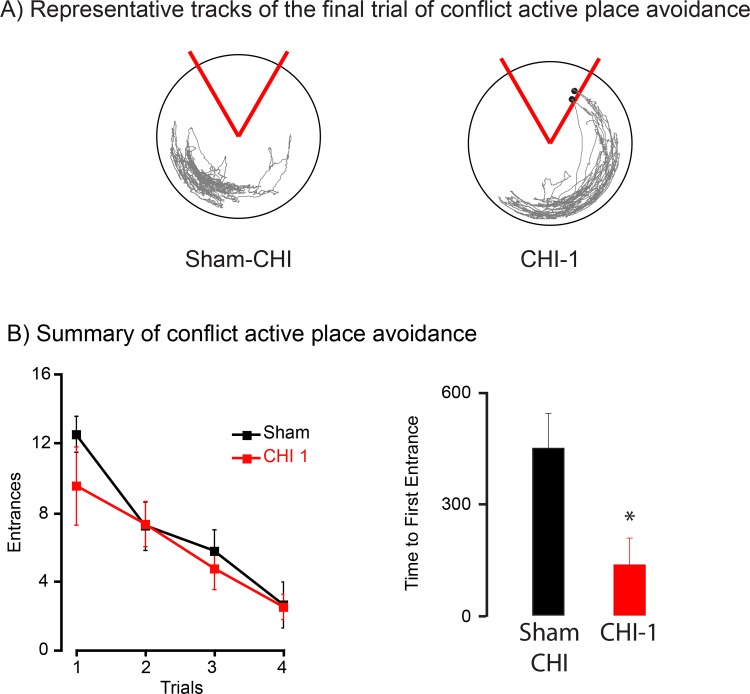
CHI-1 mice acquire, but do not retain, a new shock zone location in conflict active place avoidance. Panel A, Representative tracks from sham-CHI and CHI-1 mice in the final trial of conflict active place avoidance. The 60° stationary shock zone is shown in red. Black circles show the location of the mouse when receiving a shock. Panel B, Summary of conflict active place avoidance. On shock zone entrances during conflict active place avoidance, group and trial did not significantly interact (F_1630,30_ = 14.0, p = 0.50). The number of entrances did not significantly differ. (F_1,10_ = 0.006, p = 0.94).

Sham-CHI and CHI-1 mice were able to acquire the active place avoidance task so they were tested the next day on a conflict version of the active place avoidance task with the shock zone location rotated 180° from the previous days location (Fig 3). CHI-2 mice were not tested since they did not acquire the shock zone location on the first day of testing (Fig 2). Sham-CHI and CHI-1 had a similar number of shock zone entrances on the 4^th^ and final trial, yet they significantly differed on time to 1st entrance for the final trial (Fig 3B). These data further suggest that CHI-1 mice acquired, but did not retain the shock zone location.

In contrast, they significantly differed on time to 1st entrance for the final trial (t_10_ = 2.16, p < 0.05) (Fig 3B). These data further suggest that CHI-1 mice acquired, but did not retain, the shock zone location.

### Behavioral deficits of CHI-1 and CHI-2 mice are retained one month after injury

**Fig 4:**
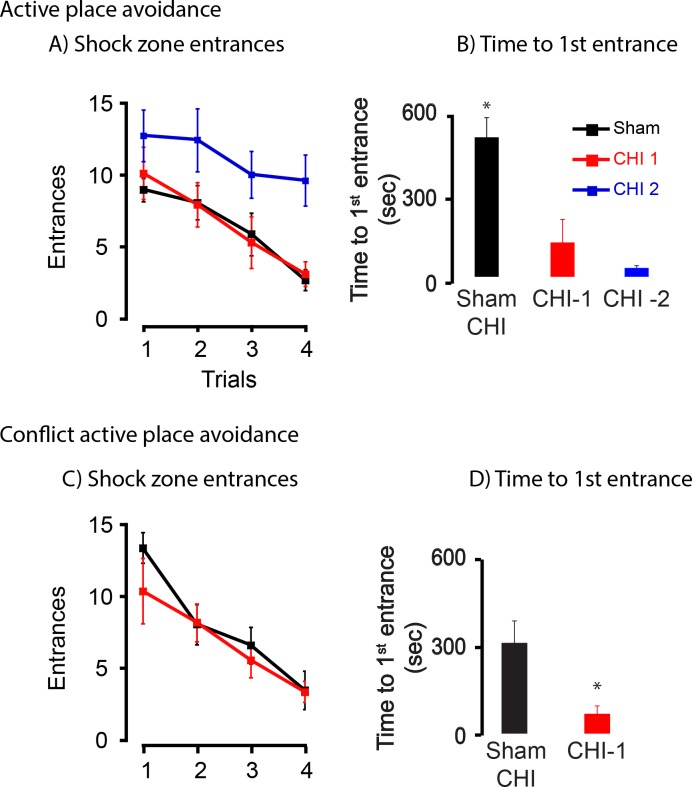
CHI-1 and CHI-2 mice retain behavioral deficits one month after injury. Panel A, Summary of active place avoidance one month after injury. Active place avoidance at one month post-injury showed a significant interaction between group and trial (F_6,51_ = 6.06, p < 0.0005). The sham-CHI and CHI-2 groups significantly differed between the sham-CHI and CHI 2 (post-hoc, p = 0.001). The CHI 1 group strongly trended toward differing from the CHI 2 group (p = 0.056). Panel B, Sham-CHI mice had a significantly longer time to 1st entrance than CHI-1 or CHI-2 mice (post hoc *p < 0.01). Panel C, At 1 month, number of entrances for conflict active place avoidance, group and trial did not statistically interact (F_1764,39_) = 0.662, p = 0.51). The two groups had a similar number of entrances (F_1,13_ = 0.136, p = 0.72). On the final trial of conflict active place avoidance, sham-CHI and CHI-1 mice had a similar number of total entrances (t_10_ = 0.3, p > 0.5), but differed significantly on time to 1st entrance (t_10_ = 2.98, *p < 0.02).

A second set of sham-CHI (n = 8), CHI-1 (n = 4) and CHI-2 (n = 8) mice tested whether behavioral deficits seen at one week were retained at one month (Fig 4). After sham-CHI or CHI, mice were returned to their home cages for one month. On the open field/habituation trial, all groups traveled a similar distance (in meters), (Sham-CHI, 45.7 ± 2.7; CHI-1, 42.2 ± 5.1; CHI-2, 46.8 ± 2.8; ANOVA, F_2,19_ = 0.37, p > 0.5). On active place avoidance, total shock zone entrances showed a significant group effect (ANOVA, F_2,19_ = 0.37, p < 0.01) (Fig 4A). The CHI-2 group had significantly more entrances that the sham-CHI or CHI-1 groups (post hoc, p < 0.01). Time to 1st entrance on the final trial of active place avoidance also had a significant group effect (ANOVA, F_2,19_ = 5.18, p < 0.02) with the sham-CHI group having more that the CHI-1and CHI-2 groups (post hoc, p < 0.05) (Fig 4A). Sham-CHI and CHI-1 mice were tested the following day on the conflict version of the active place avoidance task (Fig 4B). The sham-CHI and CHI-1 groups had a similar number of total entrances (t_10_ = 0.3, p > 0.5), but differed in time to first entrance (t_10_ = 2.98, p < 0.02). Similar to seven days after injury, these data suggest that CHI-1 animals acquired but did not retain the new shock zone entrance during conflict active place avoidance. These data also suggest that the impairments in the CHI-1 group persist one month after the injury and remain different from CHI-2 group.

Behavioral deficits may persist over time after injury, but differ in extent; therefore we directly compared behavioral parameters of each group at one week and one month. Neither the total shock zone entrances nor the time to 1st entrance at one week and one month differed between the sham-CHI, CHI-1 and CHI-2 groups (entrances, sham-CHI, t_11_ = -2.0, p >0.05; CHI-1, t_8_ = -1.9, p >0.05; CHI-2 t_11_ = 1.0, p >0.1), (time to 1st entrance, sham-CHI, t_11_ = 1.1, p >0.1; CHI-1, t_8_ = 0.55, p >0.5; CHI-2, t_11_ = -1.9, p >0.05). Thus, the behavioral deficits of the CHI-1 and CHI-2 groups at one week remained similar to the deficits observed at one month.

### CHI-1 and CHI-2 mice have differing patterns of white matter injury, but similar grey matter injury

**Fig 5:**
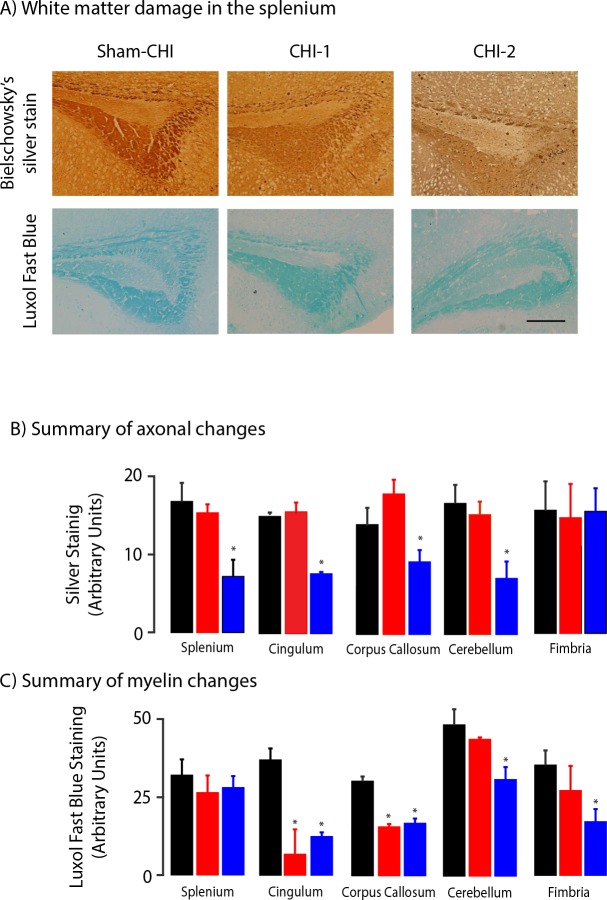
Assessment of white matter injury in CHI-1 and CHI-2 mice. Panel A, Representative photomicrographs of sagittal sections of splenium stained with Bielschowsky's modified silver stain (top), or the luxol fast blue myelin stain (bottom). The scale bar is 200μm. Panel B, Summary of changes in silver stain intensity in CHI-1 and CHI-2 mice. Panel C, Summary of myelin loss in CHI-1 and CHI-2 mice. In some brain regions, silver stain or myelin stain intensity in the CHI-1 or CHI-2 groups was significantly lower than in sham-CHI mice (post hoc, *p < 0.05).

White matter injury was assessed in parasagittal sections prepared from the brains of the Sham-CHI, CHI-1 and CHI-2 groups one month after the injury. The number of surviving axons was assessed using Bielschowsky’s modified silver stain (Fig 5A) [17]. In the splenium, there was a significant group effect for silver staining intensity (ANOVA, F_2,10_ = 5.88, p < 0.05). Silver staining intensity in the splenium of the CHI-2 group was significantly less than the CHI-1 or sham-CHI groups (post hoc, p < 0.05) (Fig 5B). A significant group effect for silver staining intensity was also observed in the cingulum, corpus callosum and cerebellum, but not in the fimbria (ANOVA, cingulum, F_2,10_ = 10.7, p < 0.01; corpus callosum, F_2,10_ = 4.4, p < 0.05; cerebellum, F_2,10_ = 5.9, p < 0.03, fimbria, F_2,10_ = 0.02, p > 0.5). Silver staining intensity of the CHI-1 group in the splenium, cingulum, corpus callosum and cerebellum was equivalent to the sham-CHI group; in contrast, staining intensity in the CHI-2 group was significantly lower (post hoc, p < 0.05). These data suggest that the CHI-2 group sustained axonal loss not seen in the CHI-1 group. Both the CHI-1 and CHI-2 groups had reduced neurofilament expression in multiple white matter regions as compared to the sham-CHI group. Neurofilament expression was similar between the CHI-1 and CHI-2 groups (S1 Fig).

Myelin content was assessed by luxol fast blue (LFB) staining (Fig 5A). LFB staining did not significantly differ among the sham-CHI, CHI-1 and CHI-2 groups in the splenium but differed significantly in the cingulum, corpus callosum, cerebellum and fimbria (ANOVA, splenium, F_2,9_ = 0.43, p > 0.5, cingulum, F_2,9_ = 17.8, p<0.002; corpus callosum, F_2,9_ = 6.0, p<0.05, cerebellum, F_2,9_ = 5.2, p<0.05; fimbria, F_2,9_ = 4.8, p < 0.05). The CHI-1 and CHI-2 groups have similar significant myelin loss in the cingulum and corpus callosum, yet only the CHI-2 group had significant myelin loss in the cerebellum and fimbria (post hoc, p < 0.05).

**Fig 6:**
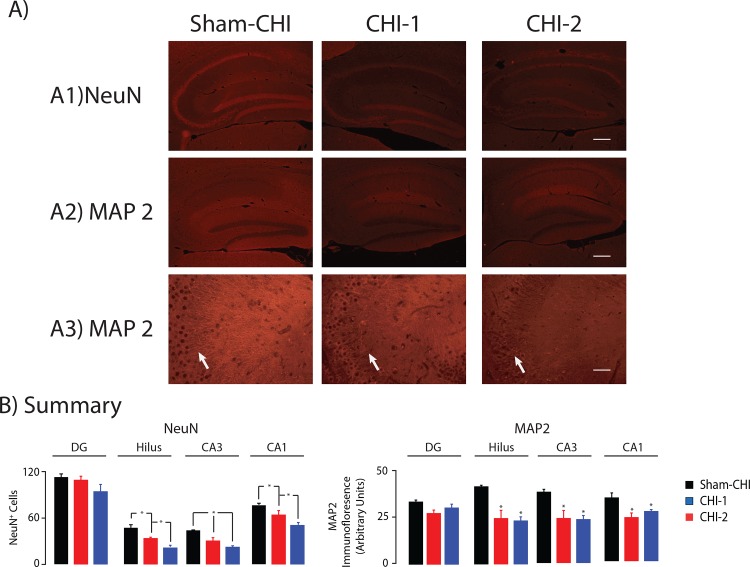
Grey matter injury in the hippocampi of CHI-1 and CHI-2 mice. Panel A, Representative photomicrographs of sagittal sections of hippocampus one month after injury stained with antibodies against NeuN (Panel A1) or MAP2 (Panels A2, A3). Panel A3 provides a higher magnification of the CA3 region of the images in Panel A2. Dendrites are fewer and more disorganized in the CHI-1 and CHI-2 groups (arrows). Panel B, Summary of changes in number of NeuN+ cells (left) in the CA3 and CA1 pyramidal cell layers, the granule cell layer of the dentate gyrus or the hilus. The number of NeuN+ cells in the CHI-2 group was significantly lower than the CHI-1 group in the hilus and the CA1 pyramidal cell layer (post hoc, p > 0.05). The CHI-1 and CHI-2 groups had a similar number of NeuN+ cells in the CA3 pyramidal cell layer of CA3; this number was significantly lower than in the sham-CHI group (post hoc p < 0.005). The CHI- 1 and CHI-2 groups had similar loss of MAP2 immunoreactivity in the hilus and the pyramidal cell layers of CA1 and CA3. The amount of MAP2 immunoreactivity was significantly lower than in the sham-CHI group (post hoc p < 0.05). Panel A3 provides a higher magnification of the CA3 region of the images in Panel A2. Dendrites are fewer and more disorganized in the CHI-1 and CHI-2 groups (arrows). Scale bar, 200μm; panels A and B; 100μm, panel C.

One month after the injury, the hippocampus ipsilateral to the impact site was examined (Fig 6). Neuronal nuclei were labeled using an anti-NeuN antibody in the sham-CHI (n = 3), CHI-1 (n = 3) and CHI-2 (n = 3) groups (Fig 6A1). There was a significant group effect on the number of NeuN^+^ cells in the pyramidal cell layer of the CA3 and CA1 and in the hilus (ANOVA, hilus, F_2,8_ = 20.9. p < 0.005; CA3, F_2,8_ = 20.6, p< 0.005, CA1, F_2,8_ = 10.6, p > 0.01) but not in the granule cell layer of the dentate gyrus (ANOVA, F_2,8_ = 0.49, p > 0.5). In the hilus, and the CA3 and CA1 pyramidal cell layers, the CHI-1 and CHI-2 group had fewer NeuN^+^ cells than the sham-CHI group (post hoc, p < 0.005). In the hilus and CA1 pyramidal cell layer, CHI-2 mice had fewer NeuN^+^ cells than the CHI-1 mice (Fig 6A2). MAP2 immunoreactivity was analyzed in the sham- CHI (n = 4), CHI-1 (n = 3) and CHI-2 (n = 3) groups. MAP2 immunoreactivity had a significant group effect in the hilus, CA3 and CA1 regions, but not in the granule cell layer of the dentate gyrus (ANOVA, hilus F_2,9_ = 11.6, p<0.001; CA3, F_2,9_ = 5.3, p< 0.05, CA1, F_2,9_ = 6.3, p < 0.05, granule cell layer, F_2,9_ = 0.8, p > 0.4). CHI-1 and CHI-2 mice had a similar loss of MAP2 expression as compared to sham-CHI mice (post hoc, p < 0.01). MAP2 positive dendrites projected orthogonally from CA3 stratum pyramidale into stratum lucidum and stratum radiatum. These dendrites were fewer and less organized in the CHI-1 and CHI-2 groups (Fig 6A3). These data suggest that CHI-1 mice had more neuronal loss than CHI-2 mice one month after injury.

### Analysis of head movement in mice during and immediately after closed head injury

**Fig 7:**
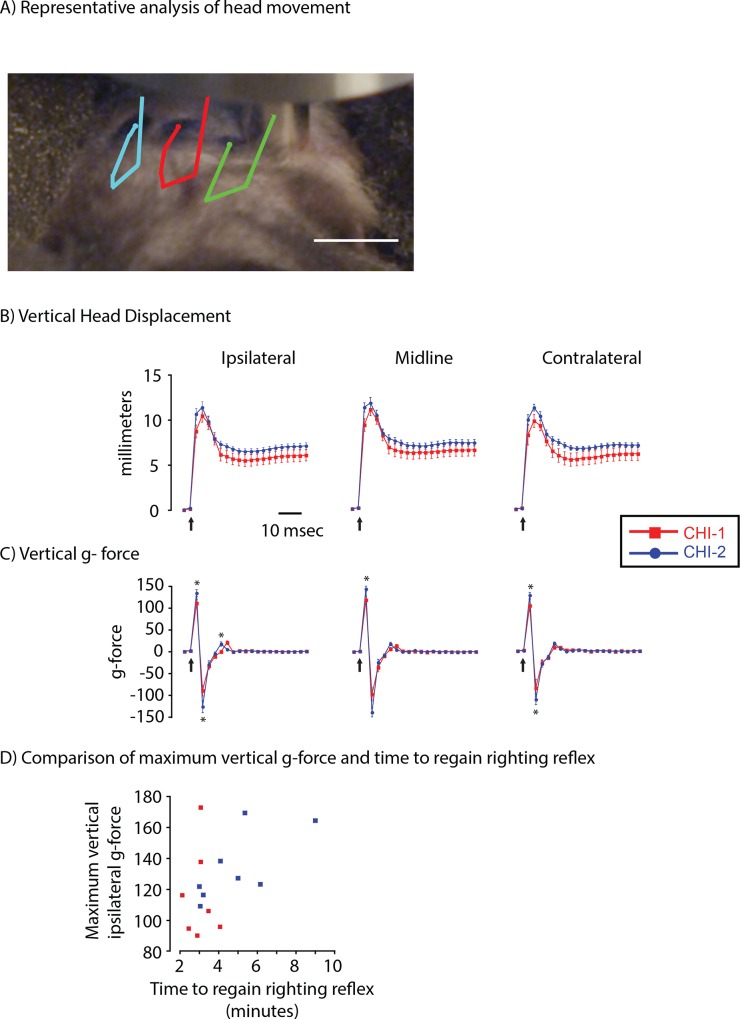
A single impact produces a heterogeneous vertical g-force. Panel A, A video still image taken at 16.8msec after impact; a time when the head was no longer moving. A color overlay shows the movement of the ipsilateral (green), midline (red) and contralateral (blue) spots after the impact. Scale bar, 10 mm. The units of g-force are newtons per kg. Panel B, Summary of high-speed video analysis of movements of spots ipsilateral, midline and contralateral to the impact site. An arrow indicates the time of the impact. Panel C, Summary of vertical g-forces computed ipsilateral, midline and contralateral to the impact site. CHI-2 mice had a significantly larger acceleration and deceleration at all three sites than CHI-1 mice (post hoc, *p<0.05). CHI-2 mice had greater rebound acceleration than CHI-1 mice (post hoc, *p<0.05). Panel D, Scatter plot of vertical g-force of individual CHI-1 and CHI-2 mice with time to regain righting reflex. The CHI-2 (**ρ** = 0.724, p = 0.05), but not CHI-1 (**ρ** = -0.468, p > 0.2) group had a significant correlation with the time needed to regain righting reflex.

A high-speed video camera recorded the motion of the heads of mice at 52.8 msec intervals before, during and after impact. Vertical, lateral and angular head motions were recorded and computed from three markers; two lateral to the impact and one located on the midline (Fig 7A). A still image from this video taken 16.8 msec after impact is shown in Fig 7A from the entire injury (S1 Video). After the injury, the mice were divided into two groups (CHI-1, n = 7, and CHI-2, n = 8) based upon duration of apnea and righting reflex (CHI-1, 2.8 ± 0.26 min; CHI-2, 4.9 ± 0.73 min; t_13_ = -2.32, p <0.05). The vertical head displacement of the CHI-1 and CHI-2 groups showed a significant effect of time with no effect of group or an interaction between group and time (Repeated values ANOVA; Group, Ipsilateral, F_1,13_ = 2.18, p = 0.16, Midline, F_1,13_ = 1.43, p = 0.25, Contralateral, F_1,13_ = 0.18, p = 0.12; Time, Ipsilateral, F_20,260_ = 159.2, p < 0.0001, Midline, F_20,260_ = 211.0, p < 0.0001, Contralateral, F_20,260_ = 173.2, p < 0.0001; Interaction, Ipsilateral, F_20,252_ = 1.28, p = 1.19, Midline, F_20,252_ = 1.19, p = 0.26, Contralateral, F_20,252_ = 1.25, p = 0.22) (Fig 7B). Vertical g-forces were computed from the vertical head displacement (Fig 7C). Vertical g-force experienced by the CHI-1 and CHI-2 groups differed significantly over time without a significant group effect. Group and time significantly interacted at the measurement points ipsilateral and midline to the impact, but not at the contralateral measurement point (Repeated values ANOVA; Group, Ipsilateral, F_1,13_ = 0.26, p = 0.62, Midline, F_1,13_ = 0.24, p = 0.63, Contralateral, F_1,13_ = 0.12, p = 0.73; Time, Ipsilateral, F_20,260_ = 159.2, p < 0.0001, Midline, F_20,260_ = 199.3, p < 0.0001, Contralateral, F_20,260_ = 105.7, p < 0.0001; Interaction, Ipsilateral, F_20,260_ = 3.47, p < 0.0001, Midline, F_20,260_ = 4.63, p < 0.0001, Contralateral, F_20,260_ = 1.25, p = 0.08). Post hoc analysis indicated that the heads of CHI-2 mice had more acceleration at 5.6 msecs and more deceleration at 8.4 msecs after injury than CHI-1 mice (Fig 7C). The CHI-2 group had a greater rebound acceleration at 16.8 msecs. The maximum ipsilateral vertical g- force of individual mice was then correlated with the restoration of righting reflex (Fig 7D). The time needed to regain righting reflex in the CHI-2 group significantly correlated with maximum vertical g-force while no significant correlation was seen with CHI-1 mice (Fig 7D).

The lateral head displacement of the CHI-1 and CHI-2 groups did not significantly differ with g-forces in both groups approximately 3 fold smaller in the lateral direction than in the vertical direction (S2 Fig). In contrast, the CHI-2 group showed a strong trend toward receiving more angular displacement and angular acceleration (post hoc, p = 0.07) (S2 Fig). This raises the possibility that angular motions contributed to the differences between the CHI-1 and CHI-2 group. A role for angular motions is consistent with the body of knowledge showing the importance of rotational movements in clinical TBI [18].

## Discussion

This study describes two injury syndromes, CHI-1 and CHI-2 that were initially separated based upon the ability to regain spontaneous breathing and righting reflex (Fig 1A). CHI-1 and CHI-2 mice had differing and long-lasting behavioral deficits. One week after injury, CHI-1 mice readily acquired a shock zone location in active place avoidance testing but had no retention of the location. CHI-2 mice neither acquired nor retained the shock zone location (Figs 2 and 3). These behavioral deficits of CHI-1 and CHI-2 mice persisted one month after injury (Fig 4). The CHI-2 group had more hippocampal and white matter damage than the CHI-1 group (Figs 5 and 6). CHI-2 mice experienced a significantly greater vertical g-force than CHI-1 mice after the impact (Fig 7). In CHI-2 mice, the amount of vertical g-force significantly correlated with time to regain righting reflex (Fig 7D). No significant correlation was seen with CHI-1 mice. Thus, post-injury apnea and the delay in the restoration of righting reflex may be valuable surrogate markers for the development of long-lasting impairments. The injury parameters used in this study consistently produced both CHI-1 and CHI-2 groups.

Measurements of head displacement were taken from tracking markers placed on the skin of the animal's head (Fig 7A, S2 Fig, S1 Video). One caveat of this analysis is that the g- forces are computed from the markers on the skin that differ from the g-forces evoked in the brain after the impact. The behavioral and histological impairments of CHI-1 and CHI-2 mice could develop from brain damage arising from compression through the skull. The mouse skull is thin and pliable, thus the impactor may have deformed the skull and the underlying brain parenchyma. Local deformation of the skull is consistent with the presence of hemorrhage underlying the impact site in the CHI-2 mice that died within 24 hours after injury. Additionally, CHI-2 mice showed a strong trend toward greater angular head movements than the CHI-1 group (S2 Fig). Finally, vertical g-force received by CHI-2 mice may translate to greater movements of the brain (Fig 7C). The use of an electromagnetic impactor makes it unlikely the two groups arose from the differences in the impact force. It is uncertain, however, what underlies the differences in the head movements. Small variations in the weight, the shape or volume of the head may underlie these differences. All of these potential mechanisms could act singly or in concert to produce the more severe deficits of the CHI-2 group.

The CHI-1 and CHI-2 groups differed in righting reflex (Fig 1A). Restoration of unassisted breathing and righting reflex occurred within seconds and minutes after injury, respectively. Apnea largely arises from shear forces at the junction of the brainstem and the forebrain[13, 19–21]. Differences in brain compression, vertical g-force or angular movements may have caused more injury in the brainstem-forebrain junction in the CHI-2 mice than in the CHI-1 mice. Acute injury in the brainstem-forebrain junction was not examined. Righting reflex has been used as an acute marker of brain injury in open head models of TBI, including the fluid percussion and controlled cortical impact models [3, 22]. This is the first description of the use of righting reflex in a closed head TBI model.

The widespread white matter injury in the CHI-2 group of mice is the likely cause of the substantial deficits on the active place avoidance task (Figs 3–5). We previously showed that rats with localized demyelination of the fimbria do not acquire the active place avoidance task [9]. The fimbria of the CHI-1 mice had similar silver and LFB staining as the sham-CHI group (Fig 5). The fimbria of the CHI-2 group may have long-lasting demyelination since they had significantly less LFB staining than the CHI-1 or sham-CHI groups. The large and long-lasting deficit of the CHI-2 group on active place avoidance may be due, in part, to selective demyelination of the fimbria.

CHI-1 mice acquired the active place avoidance task but were unable to increase time to first entrance into the shock zone (Table 1). This suggests impairment in long-term task acquisition. The hippocampus is critical in increasing time to first entrance on the active place avoidance task [16]. CHI-1 mice had significantly fewer neurons in the CA1, CA3 and hilar regions of the hippocampus than sham-CHI mice (Fig 6). The hippocampal injury found in CHI-1 mice may explain the inability of the CHI-1 group to increase time to first entrance as compared to CHI-2 mice.

Three other groups have injured C57/BL6 mice using a similar impactor as in this study. Creed, et al., and Hylin, et al., used mice of the same age and weights as this study while Mouzon, et al., used older mice [7, 8, 23]. All three groups used less force to strike the head. Injured mice in the Hylin, et al. study had similar behavioral deficits as CHI-1 mice in the active place avoidance task and lacked deficits in the Morris water maze. Mice in the Creed, et al., and the Mouzon, et al., studies were not tested on active place avoidance but lacked behavioral deficits on the Morris water or the closely related Barnes maze. The pattern of behavioral deficits suggests that the mice injured in the Creed, Hylin and Mouzon studies most closely resemble the CHI-1 mice of this study. This is consistent with the lower force of the impactor to strike the head.

CHI-2 mice required cardiopulmonary resuscitation with supplementation of 100% O_2_ to survive until unassisted breathing was restored. Supplementation of 100% O_2_ can increase oxidative stress that can injure the brain[24]. The possibility exists that some deficits in CHI-2 mice arise from either anoxia or from the increased oxidative stress from the O_2_ supplementation. Prolonged apnea frequently accompanies clinical TBI and ventilation with O_2_ is a standard procedure for such patients[25]. Thus, the breathing support received by CHI-2 mice mimics the treatment of brain-injured patients with prolonged apnea.

## Supporting Information

S1 Fig Analysis of neurofilament expression Panel A, Representative photomicrographs of sagittal sections of cerebellum stained with an antibody against neurofilament. Scale Bar, 200 μm Panel B, Summary of the neurofilament immunoreactivity in sham-CHI (n = 5) CHI-1 (n = 4) and CHI-2 (n = 5) mice. Neurofilament expression significantly differed among three groups in the cingulum, corpus callosum, and splenium (ANOVA, Cingulum, F_2,11_ = 13.2, p<0.002; Corpus Callosum, F_2,11_ = 9.0, p<0.005; Splenium, F_2,11_ = 5.6, p<0.05) but not in the fimbria (ANOVA, F_2,11_ = 2.0, p>0.2). CHI-2 mice had less neurofilament immunoreactivity than CHI-1 in corpus callosum, splenium and cingulum (post hoc, p< 0.05). Both CHI-1 and CHI-2 had less neurofilament expression than sham-CHI (post hoc, p < 0.05). In fimbria, CHI-2, but not CHI-1 mice had reduced neurofilament expression compared to sham-CHI (post hoc, p < 0.05).
(EPS)

S2 Fig Lateral and angular head movement after impact. Panel A, Summary of lateral head displacement measured ipsilateral, midline and contralateral to the impact site. CHI-1 and CHI-2 mice had displacement at all three sites. Panel B, Summary of lateral g-forces computed ipsilateral, midline and contralateral to the impact site. CHI-2 mice trended toward greater lateral head accelerations than CHI-1 mice (post hoc, p = 0.07). Panel C, Angular displacement of the head during impact. There was no significant difference between CHI-1 and CHI-2 mice. Panel D, Summary of angular g-forces. There was no significant difference between CHI-1 and CHI-2 mice.
(EPS)

S1 Video High-speed video recording of the head movement of a mouse before, during and after a blunt impact. Fig 7A shows a still picture from the same video. The entire time elapsed in the video is 56 msec. Scale bar, 10 mm.
(MOV)
